# Preliminary Screening for Hereditary Breast and Ovarian Cancer Using an AI Chatbot as a Genetic Counselor: Clinical Study

**DOI:** 10.2196/48914

**Published:** 2024-11-27

**Authors:** Ann Sato, Eri Haneda, Yukihiko Hiroshima, Hiroto Narimatsu

**Affiliations:** 1 Department of Genetic Medicine Kanagawa Cancer Center Yokohama, Kanagawa Japan; 2 Graduate School of Health Innovation Kanagawa University of Human Services Kawasaki, Kanagawa Japan; 3 Department of Cancer Genome Medicine Kanagawa Cancer Center Yokohama, Kanagawa Japan; 4 Advanced Cancer Therapy Division Kanagawa Cancer Center Research Institute Yokohama, Kanagawa Japan; 5 Cancer Prevention and Cancer Control Division Kanagawa Cancer Center Research Institute Yokohama, Kanagawa Japan

**Keywords:** hereditary cancer, familial cancer, IBM Watson, family history, medical history, cancer, feasibility, social network, screening, breast cancer, ovarian cancer, artificial intelligence, AI, chatbot, genetic, counselling, oncology, conversational agent, implementation, usability, acceptability

## Abstract

**Background:**

Hereditary breast and ovarian cancer (HBOC) is a major type of hereditary cancer. Establishing effective screening to identify high-risk individuals for HBOC remains a challenge. We developed a prototype of a chatbot system that uses artificial intelligence (AI) for preliminary HBOC screening to determine whether individuals meet the National Comprehensive Cancer Network *BRCA1/2* testing criteria.

**Objective:**

This study’s objective was to validate the feasibility of this chatbot in a clinical setting by using it on a patient population that visited a hospital.

**Methods:**

We validated the medical accuracy of the chatbot system by performing a test on patients who consecutively visited the Kanagawa Cancer Center. The participants completed a preoperation questionnaire to understand their background, including information technology literacy. After the operation, qualitative interviews were conducted to collect data on the usability and acceptability of the system and examine points needing improvement.

**Results:**

A total of 11 participants were enrolled between October and December 2020. All of the participants were women, and among them, 10 (91%) had cancer. According to the questionnaire, 6 (54%) participants had never heard of a chatbot, while 7 (64%) had never used one. All participants were able to complete the chatbot operation, and the average time required for the operation was 18.0 (SD 5.44) minutes. The determinations by the chatbot of whether the participants met the *BRCA1/2* testing criteria based on their medical and family history were consistent with those by certified genetic counselors (CGCs). We compared the medical histories obtained from the participants by the CGCs with those by the chatbot. Of the 11 participants, 3 (27%) entered information different from that obtained by the CGCs. These discrepancies were caused by the participant’s omissions or communication errors with the chatbot. Regarding the family histories, the chatbot provided new information for 3 (27%) of the 11 participants and complemented information for the family members of 5 (45%) participants not interviewed by the CGCs. The chatbot could not obtain some information on the family history of 6 (54%) participants due to several reasons, such as being outside of the scope of the chatbot’s interview questions, the participant’s omissions, and communication errors with the chatbot. Interview data were classified into the following: (1) features, (2) appearance, (3) usability and preferences, (4) concerns, (5) benefits, and (6) implementation. Favorable comments on implementation feasibility and comments on improvements were also obtained.

**Conclusions:**

This study demonstrated that the preliminary screening system for HBOC using an AI chatbot was feasible for real patients.

## Introduction

The rapid adoption of digital health tools has significantly enhanced health care efficiency and quality [1]. Chatbots enable communication with patients. Their use includes delivering remote health services (eg, patient support, education, and health promotion) and providing administrative assistance (eg, managing routine tasks and supporting research). One key advantage of chatbots is resource optimization. By handling routine tasks, chatbots let health care professionals (HCPs) focus on clinical duties. Chatbots standardize health care and expand access, increasing patient opportunities for appropriate services, even in regions with limited medical staff [2]. In genetic medicine, which requires specialized expertise, chatbots can collect family medical history and assist HCPs in communicating with at-risk relatives [3,4]. However, practical implementation in genetic medicine remains limited, particularly in Japan. We developed a chatbot for preliminary screening of hereditary breast and ovarian cancer (HBOC) [5] and conducted a clinical study to validate its feasibility and potential usefulness in a clinical setting with real patients.

## Methods

### Overview

The chatbot system [5] was planned to be tested on 10 patients to estimate its medical accuracy and ability to perform preliminary screening, such as obtaining correct medical and family histories and assessing the risk of HBOC according to the National Comprehensive Cancer Network guidelines. The system uses Watson, IBM’s cognitive computing service. Preoperation questionnaires and postoperation interviews validated data on the system’s usability and acceptability. Additional details are provided in Multimedia Appendix 1.

### Ethical Considerations

This study was approved by the institutional review board of Kanagawa Cancer Center (2020 - epidemiology 78). Written informed consent was obtained from all participants. An ID was assigned for each participant, and personal information was excluded. The correspondence table between individuals and IDs is stored in a secure database that is not connected to the internet, and the database terminal is stored under double-locked security. We provided each participant with JP ¥2000 (~US $15) as an honorarium.

## Results

A total of 11 participants enrolled between October and December 2020 (Table 1). All participants completed the chatbot operation (Multimedia Appendix 2). Preliminary screening results are shown in Table 1. Qualitative data from postoperation interviews are shown in Table 2, with key comments in Multimedia Appendix 3.

**Table 1:** Participant characteristics and result.

Variable						Participants (n=11)^a^
**Demographic factors**						
	**Age (years),** **median (range)**				52 (35-67)	
	**Education^b^, n (%)**					
		Low		0 (0)		
		Middle		2 (18)		
		High		9 (82)		
	**Familiar with chatbots, n (%)**					
		Yes		4 (36)		
		No		6 (55)		
		I am not sure		1 (9)		
	**Have used a chatbot, n (%)**					
		Yes		4 (36)		
		No		7 (64)		
	**Reaction to new technology, n (%)**					
		Eager		6 (55)		
		Overwhelmed		4 (36)		
		It depends		1 (9)		
	**Any cancers or past history of cancers, n (%)**					
		**Yes**		10 (91)^c^		
			Breast cancer	7 (64)		
			Ovarian cancer	1 (9)		
			Stomach cancer	1 (9)		
			Double cancer	1 (9)		
		**No**		1 (9)		
**Preliminary screening results^d^**						
	**Eligibility of the** ***BRCA1/2*** **testing criteria**					
		**Met**		7 (64)		
			Consistent with experts’ evaluations based on the same data	7 (64)		
			Consistent with experts’ evaluations based on information gathered by the CGCs^e^ before this study	7 (64)		
		**Unmet**		4 (36)		
			Consistent with experts’ evaluations based on the same data	4 (36)		
			Consistent with experts’ evaluations based on information gathered by the CGCs before this study	1 (9)		
			Inconsistent with experts’ evaluations based on information gathered by the CGCs before this study	3^f^ (27)		

^a^Participants who consecutively visited the Department of Genetic Medicine at the Kanagawa Cancer Center and met the selection criteria were enrolled in this study. The inclusion criteria were male or female individuals aged ≥30 years during inclusion in this study, with a history of breast and/or ovarian cancer; healthy individuals with family histories of breast and/or ovarian cancer; and those who had been interviewed by CGCs and had provided their family histories to CGCs at the Kanagawa Cancer Center before the start of the study. Individuals with difficulty communicating in Japanese, those who could not operate the tablet (text input, tapping, and scrolling, among others), and those with difficulty completing the questionnaire were excluded from this study. The questionnaire was administered to each participant before they used the chatbot system (Multimedia Appendix 1). The participants accessed this system using the tablet we provided. One or two CGCs observed each participant operating the chatbot system. The CGCs responded to any questions from the participants. We measured the time required for each participant to complete the chatbot operation. Two CGCs (authors AS and EH) compared the medical and family histories collected via the chatbot with those previously obtained by CGCs immediately after the chatbot operation was completed. If a discrepancy was found, it was clarified whether it resulted from a system error or participant’s omissions. The accuracy of preliminary screening with this system was evaluated by experts, including a doctor specializing in genetic medicine (author HN) and 2 CGCs, based on the National Comprehensive Cancer Network guidelines. After the chatbot operation, we conducted a semistructured interview, which lasted approximately 30 minutes. The interview focused on opinions and impressions about this system and was conducted once, according to the interview guide (Multimedia Appendix 1).

^b^Low: middle school graduate; middle: high school graduate; and high: vocational school, junior college, college, or graduate school graduate.

^c^Includes 1 individual diagnosed with hereditary breast and ovarian cancer.

^d^The median time for the medical and family history interview by this system was 17.0 minutes.

^e^CGC: certified genetic counselor.

^f^Based on information gathered by the CGCs before this study, 3 of the 4 participants who did not meet the *BRCA1/2* testing criteria via chatbot actually met the National Comprehensive Cancer Network criteria. Two discrepancies were due to communication errors with the chatbot, where family and medical histories were incorrectly entered as follows: one involved peritoneal cancer being entered as “other cancers” instead of “ovarian cancer,” and another where only past medical history was entered, omitting the patient’s current breast cancer. The third discrepancy was due to the lack of information regarding triple-negative breast cancer, which was excluded from the scope of the chatbot’s interview questions.

**Table 2:** Details of the qualitative data classification obtained from the post–chatbot operation interviews^a^.

Category, subcategory, and detail classification						Participants who had comments, n
**Feature**						
	**Character design**					
		*Friendly character design*			3	
		*Suggestion for improvement*			1^b^	
	**Tone and flow of speech**					
		*Appropriate*			3	
		*Suggestion for improvement*			8	
			Wording	—^c^		
			Question design	—		
			More words than necessary	—		
			Fewer words than necessary	—		
	**Ease of operation**					
		*User-friendly operability*			5	
		*Suggestion for improvement*			10	
			Confirmation of input information	—		
			Large input volume	—		
			Input method	—		
	**System to increase completion rates**					
		*Suggestion for improvement*			11	
			Nonessential feature	—		
			Nonexistent features	—		
	**Correction of input errors**					
		*Suggestion for improvement*			2^d^	
**Appearance**						
	**Avatar images**					
		*Familiar avatar image*			2	
		*Suggestion for improvement*			1^e^	
	**Overall screen visibility**					
		*Easy-to-read design*			8	
		*Suggestion for improvement*			4	
			Graphic size	—		
			Image size	—		
			Color scheme	—		
**Usability and preferences**						
	**Usability and preferences**					
		*General comment about usability*			7	
		*Excellent usability of user interface*			8	
		*Depends on age*			2	
		*Suggestion for improvement*			4	
			Graphical user interface	—		
			Expression range of the chatbot responses	—		
			Other	—		
**Concerns**						
	Security management of information				3	
	Poor network condition				2	
**Benefits**						
	Superior ease of use				5	
	Effective use of waiting time				5	
	Reduced time required for medical interview				2	
	Overall efficiency of the clinical experience				1	
	Expanded preliminary screening coverage				1	
	Research use				1	
	Safety in the COVID-19 pandemic				1	
**Implementation**						
	**Excellent implementability**				8	
	**Considerations during operation**					
		*Suggestion for improvement*			6	
			In-person support	—		
			Prior explanation	—		
			Voice guidance	—		
			Feedback on screening results	—		

^a^We transcribed the interview data verbatim and coded them accordingly. We established codes in which 1 code corresponded to 1 opinion. Subsequently, we categorized each code according to the structure of the questionnaire. The codes in each category were classified into subcategories through discussion between 2 experts (authors AS and HN). Classification into subcategories was based on the content of the responses, such as whether participants were describing the strength of the system or points needing improvement.

^b^A code described character personalities.

^c^Not applicable.

^d^Codes described nonessential features.

^e^A code described a nonessential feature.

## Discussion

This study demonstrated that implementing the chatbot for preliminary HBOC screening is feasible in a clinical setting. Two factors support this conclusion. First, the chatbot achieved a 100% completion rate, higher than previous studies [4]. Most participants reported excellent usability in the qualitative interviews, suggesting that the system’s interface positively influenced completion. Despite limited experience, participants successfully completed the operation, indicating its accessibility regardless of technological literacy. It could be adapted for both patients visiting hospitals and healthy individuals eligible for cancer screening. Previous studies have confirmed that chatbots can effectively enhance cancer screening by automating tasks and increasing participation rates [6,7]. Participant interviews indicate that there is potential to provide opportunities for genetic medicine to patients living in remote areas or those who experience anxiety regarding in-person consultations. If the system can serve a broader patient population, it can not only act as a substitute for expert-led preliminary screening and quality standardization but also enhance accessibility to genetic medicine. Second, the chatbot accurately determined participants’ eligibility for *BRCA1*/2 testing. Given the medical accuracy maintained in the 2 phases of our study, the findings support clinical application, although further research with diverse medical and family histories is needed for validation.

Based on the interviews, we identified areas for improvement. Discrepancies between the medical and family histories reported by the CGCs and those recorded using the chatbot suggest inadequacies in the chatbot’s interview, which could lead to missing individuals at high risk for HBOC. These discrepancies arose from incomplete questions from the chatbot, participants’ omissions, or communication errors. Thus, the chatbot’s questions should be extended to include all information gathered during the CGC interviews. Participants also reported difficulty viewing the family tree image, indicating that larger images and text or voice confirmations could improve accuracy. Furthermore, inappropriate wording in the chatbot’s responses may have led to communication errors, highlighting the need to review the chatbot’s language. We are currently improving the system.

We could not evaluate the chatbot’s impact on patient visit behavior because this study excluded physician feedback. Future research should evaluate whether the chatbot increases genetic counseling and testing rates.

This study suggests that chatbots can effectively gather diverse information. Previous research has shown that chatbots enhance communication [3,8-10]. Therefore, chatbots could be used for routine medical tasks beyond preliminary screenings, potentially improving HCPs’ effectiveness and streamlining workflow.

## Appendix

Multimedia Appendix 1 Supplementary methods.

Multimedia Appendix 2 Supplementary results.

Multimedia Appendix 3 Supplementary qualitative data.

## Abbreviations

AI: artificial intelligence
CGC: certified genetic counselor
HBOC: hereditary breast and ovarian cancer
HCP: health care professional

## Data Availability

The datasets generated or analyzed during this study are not publicly available due to restrictions imposed by informed consent from participants in this study, which has been approved by the institutional review boards of the Kanagawa Cancer Center.
