# Supplementary material for: Preliminary Screening for Hereditary Breast and Ovarian Cancer Using an AI Chatbot as a Genetic Counselor: Clinical Study
Source: J Med Internet Res. 2024 Nov 27;26:e48914. doi: 10.2196/48914 (PMC11635313; doi:10.2196/48914)
Supplement: Multimedia Appendix 1 [file jmir_v26i1e48914_app1.docx]

## Multimedia Appendix 1.

## Supplementary Methods

This study was approved by the institutional review board of Kanagawa Cancer Center (2020 - epidemiology 78), and all participants provided written informed consent.

### Chatbot

We set up a persona for the chatbot: a CGC, referred to as the “AI,” to ensure that the patients could interact with a familiar entity. The gender of the AI was set to female, as more CGCs in Japan are female than male. The age of the AI was set to be 20s, as AIs equal to or younger than patients are considered more familiar. The image of the LINE interface is shown in Figure 1. We designed this system to be operated by patients on their first visit to a hospital. Therefore, information regarding medical and family histories that would be difficult to obtain from patients on their first face-to-face visit was excluded from the scope of the chatbot’s interview questions. This included information about breast cancer subtypes and family history of distant relatives, including half-siblings of parents, siblings of grandparents, and great-grandparents. After obtaining the participant’s medical history, the chatbot asks for a family structure and family history in the following order: children, grandchildren, siblings, nieces and nephews, half-siblings, parents, aunts and uncles, cousins, and grandparents. Participants with two or more siblings can enter sibling information into the system in any order, regardless of their ages. Similarly, participants with two or more nephews, nieces, aunts, uncles, or cousins can enter their information in any order. The target relative is clearly indicated on the family tree image for each question (Figure 2). The subjects whose medical and family histories have been completed are highlighted in red, enabling the operator to confirm whose data entries have been completed. We placed a button labeled “Tips” on the screen that can be clicked to obtain information about genetic medicine to keep the user interested in the operation and increase the completion rate of the chatbot operations.

Figure S1. The image of the LINE interface


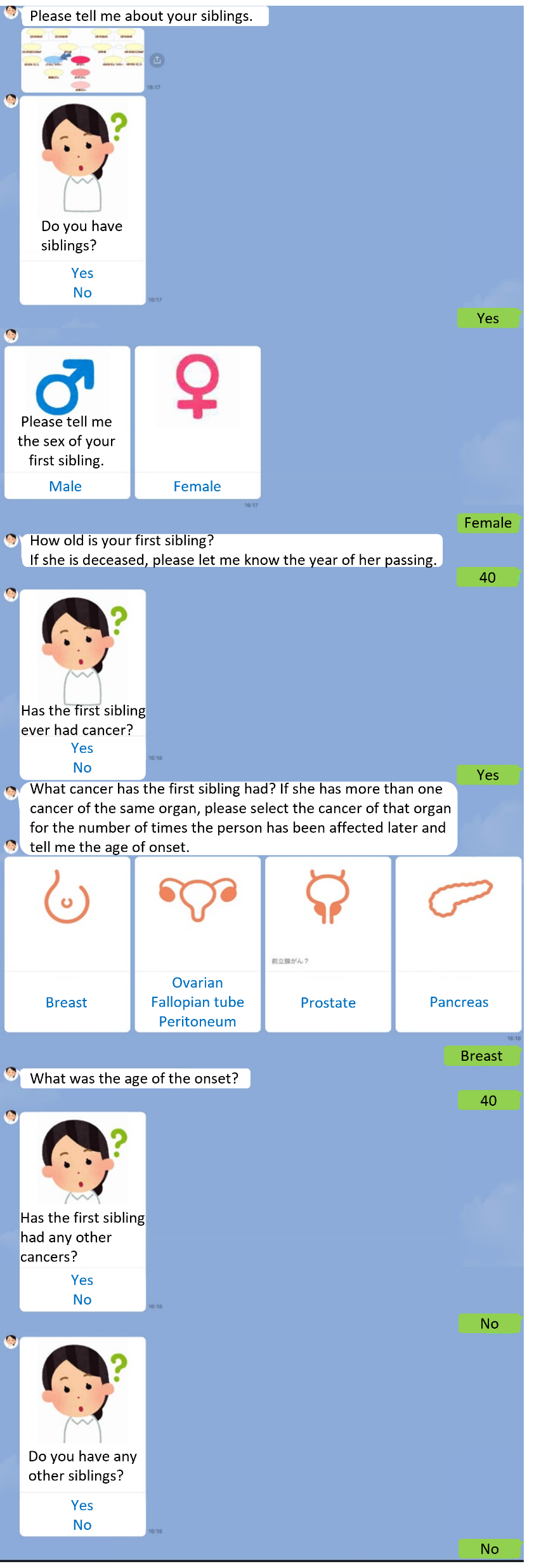


The white speech bubbles on the left side are the chatbot’s statements, and the green speech bubbles on the right side are the user’s statements. The image of the family tree that shows whose data the user is entering, located under the first white balloon from the top, can be tapped for a full-screen view. Questions from the chatbot can be answered by typing directly into the keyboard or by tapping the blue text choices in the white speech bubbles. In addition to the options shown in the screenshot (breast, ovarian, fallopian tube, peritoneal, prostate, and pancreatic cancer), “other cancers” and “don’t know” are also available as options for the type of cancer. These options are displayed by sliding the user’s finger across the area of the illustrated organs.

Figure S2. The target relative is clearly indicated on the family tree image for each question


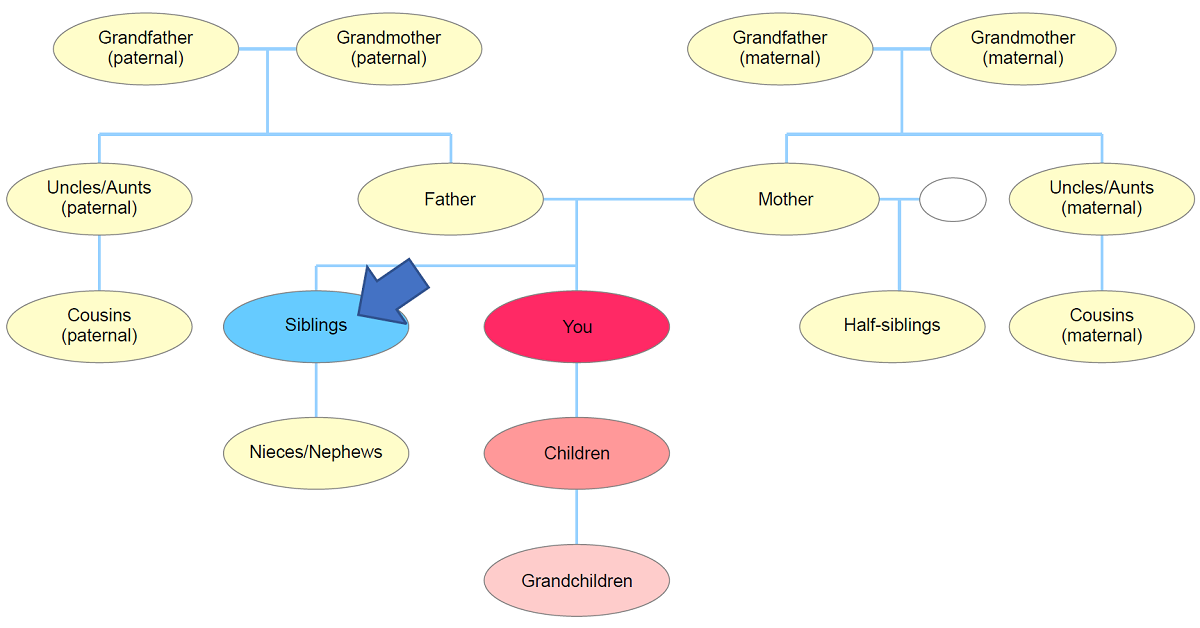


### Participants

Participants who consecutively visited the Department of Genetic Medicine at Kanagawa Cancer Center and met the selection criteria were enrolled in this study. The inclusion criteria were males or females aged ≥30 years during inclusion in this study, with a history of breast and/or ovarian cancer; healthy individuals with family histories of breast and/or ovarian cancer; and those who had been interviewed by CGCs and had provided their family histories to CGCs at Kanagawa Cancer Center before the start of the study. Individuals with difficulty communicating in Japanese, those who could not operate the tablet (text input, tapping, and scrolling, among others), and those with difficulty completing the questionnaire were excluded from this study. We provided each participant with 2,000 yen (approximately 15 US dollars) as an honorarium.

### Procedure

The questionnaire was administered to each participant before they used the chatbot system (Table 1). We designed this questionnaire according to a previous study by Schmidlen et al. *(ref: Schmidlen T, Schwartz M, DiLoreto K, Kirchner HL, Sturm AC. Patient assessment of chatbots for the scalable delivery of genetic counseling. J Genet Couns. 2019 Dec;28(6):1166-1177.)*. The participants accessed this system using the tablet we provided. One or two CGCs observed each participant operating the chatbot system. The CGCs responded to any questions from the participants. We measured the time required for each participant to complete the chatbot operation. Two CGCs (authors AS and EH) compared the medical and family histories collected via the chatbot with those previously obtained by CGCs immediately after the chatbot operation was completed. If a discrepancy was found, it was clarified whether it resulted from a system or participants’ omissions. The accuracy of preliminary screening with this system was evaluated by experts, including a doctor specializing in genetic medicine (author HN) and the two CGCs, based on the NCCN guidelines. After the chatbot operation, we conducted a semi-constructive interview survey, which lasted approximately 30 min. The interview survey focused on opinions and impressions about this system and was conducted once, according to the interview guide (Table 2).

Table S1. The questionnaire conducted on each participant before they used the chatbot system

| 1. Please choose the option that applies to your most recent educational background. |
| --- |
| ①Middle school graduate  ②High school graduate  ③Vocational school graduate  ④Junior college graduate  ⑤College graduate  ⑥Graduate school graduate  ⑦Other (　　　　　　　） |
| 1. We would like to ask about your knowledge and operating experience with chatbots. |
| ①Do you know what a chatbot is?  □　I know.  □　I don’t know.  □　I don’t know much about it.  ②Have you ever actually used a chatbot? (e.g., inquiries at customer support, among others)  □　I have used it before.  □　I have never used it.  □　I don’t know if I have ever used it. |
| 1. We would like to ask about your knowledge and operating experience of computers. |
| ①Do you have a computer (desktop/laptop)?  □　Yes.  □　No.  ②Do you have a smartphone?  □　Yes.  □　No.  ③Do you have a tablet computer?  □　Yes.  □　No.  ④How do you feel about using new technologies?  □　I would like to use it.  □　I’m not comfortable using it.  □　Depends on the situation. |

Table S2. The interview guide for the interview survey conducted after the chatbot operation

| 1. About features |
| --- |
| Examples of questions:  “How was the name, colloquial expression, flow of conversation, ease of operation/input, and tips, among others?”  “What features would you consider important when developing the chatbot?” |
| 1. About appearance |
| Examples of questions:  “How was the avatar image and screen visibility, among others?”  “What kind of appearance would you consider important when developing the chatbot?” |
| 1. About usability and preferences |
| Examples of questions:  “Do you like the usability and design, among others?”  “When developing the chatbot, how do you think it could be made more user-friendly?” |
| 1. About concerns |
| Examples of questions:  “Were there any concerns for you about the chatbot?”  “What concerns can you think of?” |
| 1. About benefits |
| Examples of questions:  “What do you think are the benefits of implementing this system in clinical practice?” |
| 1. About implementation |
| Examples of questions:  “Are there any considerations or innovations that you think are necessary for its use in clinical practice?” |

### Interview data analysis

We transcribed the interview data verbatim and coded them accordingly. We established codes in which one code corresponded to one opinion. Subsequently, we categorized each code according to the structure of the questionnaire. The codes in each category were classified into subcategories through discussion between two experts (authors AS and HN). Classification into subcategories was based on the content of the responses, such as whether participants were describing the strength of the system or points needing improvement.
