# Supplementary material for: Preliminary Screening for Hereditary Breast and Ovarian Cancer Using an AI Chatbot as a Genetic Counselor: Clinical Study
Source: J Med Internet Res. 2024 Nov 27;26:e48914. doi: 10.2196/48914 (PMC11635313; doi:10.2196/48914)
Supplement: Multimedia Appendix 2 [file jmir_v26i1e48914_app2.docx]

## Multimedia Appendix 2.

## Supplementary Results

Table S1. Differences between the medical history obtained by the CGC and that using the chatbot

| **Primary factor** | **Participant number** | **Details** |
| --- | --- | --- |
| **Omissions** |  |  |
|  | # 8 | The participant had simultaneous bilateral breast cancer; however, only a history of unilateral breast cancer was entered. |
|  | # 10 | The participant had a history of malignant colorectal polyps but entered no history of cancer. |
| **Communication errors with the chatbot** |  |  |
|  | # 6 | Even though the participant was undergoing treatment for breast cancer, she interpreted the cancer she was currently being treated for as not being included in her medical history and entered no cancer history. |

Table S2. Information on the new family history and family composition obtained using the chatbot

| **Type of information** | **Participant number** | **Relationship^a^** | **Details** |
| --- | --- | --- | --- |
| **Information on new family history provided by the chatbot** |  |  |  |
|  | # 1 | Paternal uncle | The participant recalled that the person had a history of prostate cancer and entered it. |
|  | # 6 | Maternal cousin | The participant recalled that the person had a history of breast cancer and entered it. |
|  | # 11 | Maternal uncle | The participant recalled that the person had a history of some cancer and entered it. |
| **Information on family composition complemented by the chatbot** |  |  |  |
|  | # 1 | Paternal uncle | 1 additional member. |
|  |  | Paternal aunts | 3 additional members. |
|  |  | Paternal cousins | 4 additional members. |
|  |  | Maternal cousins | 3 additional members. |
|  | # 3 | Paternal cousins | 8 additional members. |
|  |  | Maternal cousins | 6 additional members. |
|  | # 6 | Maternal uncles | 2 additional members. |
|  |  | Maternal aunt | 1 additional member. |
|  |  | Maternal cousins | 7 additional members. |
|  | # 7 | Half-siblings | 2 additional members. |
|  | # 11 | Paternal cousin | 1 additional member. |
|  |  | Maternal cousins | 6 additional members. |
| ^a^The relationship of the subject to the participant of the corresponding case number. | | | |

Table S3. Differences between the family history obtained by the CGC and that using the chatbot

| **Primary factor** | **Participant number** | **Relationship^a^** | **Details** |
| --- | --- | --- | --- |
| **Outside of the scope of the chatbot’s interview questions** |  |  |  |
|  | # 3 | Father’s half-siblings | Four individuals were out of the scope of the chatbot’s interview questions. |
|  |  | Father’s half-sibling’s child | One individual was out of the scope of the chatbot’s interview questions. |
|  |  | Mother’s half-siblings | Two individuals were out of the scope of the chatbot’s interview questions. |
|  | # 10 | Maternal grandfather’s siblings | Two individuals were out of the scope of the chatbot’s interview questions. |
|  |  | Maternal grandmother’s siblings | Seven individuals were out of the scope of the chatbot’s interview questions. |
|  |  | Great-grandparents | Two individuals were out of the scope of the chatbot’s interview questions. |
|  | # 11 | Father’s half-siblings | Two individuals were out of the scope of the chatbot’s interview questions. |
|  |  | Father’s half-sibling’s child | One individual was out of the scope of the chatbot’s interview questions. |
| **Omissions** |  |  |  |
|  | # 4 | Paternal cousin | The participant’s omissions of the information for five individuals. |
|  | # 9 | Paternal uncle | The participant’s omissions of the information for one individual. |
|  | # 10 | Maternal uncle | The participant’s omissions of the information for one individual. |
|  | # 11 | Maternal uncle or aunt | The participant’s omissions of the information for one individual. |
| **Communication errors with the chatbot** |  |  |  |
|  | # 1 | Sister | The family history of peritoneal cancer was entered as other cancers instead of as the ovarian cancer group. |
|  | # 10 | Paternal cousins | The participant mistakenly thought she was being asked about her father’s cousins instead of her paternal cousins; therefore, information for three paternal cousins was not entered. |
|  |  | Maternal cousins | The participant mistakenly thought she was being asked about her mother’s cousins instead of her maternal cousins; therefore, information for two maternal cousins was not entered. |
| ^a^The relationship of the subject to the participant of the corresponding case number. | | | |
