# Supplementary material for: Preliminary Screening for Hereditary Breast and Ovarian Cancer Using an AI Chatbot as a Genetic Counselor: Clinical Study
Source: J Med Internet Res. 2024 Nov 27;26:e48914. doi: 10.2196/48914 (PMC11635313; doi:10.2196/48914)
Supplement: Multimedia Appendix 3 [file jmir_v26i1e48914_app3.docx]

## Multimedia Appendix 3.

## Supplementary Qualitative Data

#### Category: Feature

This category was classified into the following subcategories: persona setting, tone and flow of speech, ease of operation, features to prevent boredom (i.e., Tips), and methods to correct input errors.

Three participants commented that the chatbot’s persona was friendly. One participant commented that a character without gender is more preferable so that the unique characteristics of the AI can be brought out.

Regarding the tone of the chatbot, three participants commented that the tone and flow of speech of the chatbot were appropriate, while five participants suggested improvements in the wording that would be more communicative. As an area for improvement, two participants commented that when asked about the family history of blood relatives that may exist in more than one person, they were unsure whether the order of entry should be in the order of age or the order in which they are thought of. One participant suggested improvements regarding the overall design of the questions to obtain more accurate information.

*“It is easier to stay focused on the people with cancer and ask about their medical histories. If you ask each person individually, I wonder how long it will go. So, it would be quicker and more intuitive to show a diagram and ask, ‘Have any of these people had cancer?’ If it were on paper, I would just encircle the answers and be done with it, but with this questionnaire, I am asked the information of each of the relatives, which I thought would take a lot of time.”*

Regarding the ease of operation, five participants suggested that this system had user-friendly operability. As points to be improved, eight participants commented that the image of the family tree was relatively small to confirm what they had finished entering, and four participants commented that the amount of input required was excessive. Among them, one participant expressed the following opinions:

*“Younger people probably prefer this system, but I think elderly people would not prefer this system. I guess it depends on whether they use their smartphone on a daily basis. I would prefer to write on paper by hand. On a tablet, the questions keep flowing. I sometimes worry about whether I am making any mistakes because I cannot check what I’ve entered in the earlier part of the conversation with the chatbot.”*

When we asked for feedback on the “Tips,” four participants commented that they had not noticed their existence, and seven participants commented that they did not feel that “Tips” were needed.

#### Category: Appearance

This category was classified into the following two subcategories: avatar images and overall screen visibility.

Regarding the avatar images, two participants commented that the images were friendly, and one participant commented that they were not necessary.

Regarding the overall screen visibility, eight participants commented that the screen was designed to be easy to read. Meanwhile, four participants commented on points to improve, which included the text that was relatively small for older adults to operate and the large avatar image that made it difficult to see the previous question without scrolling.

#### Category: Usability and preferences

General comments were obtained from seven participants regarding the usability of the system and user preferences. Eight participants commented on the excellent usability of LINE. Specifically, they expressed the following opinions:

*“I use LINE on a daily basis and am familiar with its operation, so it was basically easy to use without any resistance, and I felt no particular discomfort.”*

*“I wonder how it is for older people, but there are many people in their 60s and 70s who use LINE now, so it depends on the person.* *Some older people have difficulty seeing or writing, so being able to do it by touch would be convenient.”*

Two participants commented that the usability depends on the age of the operator. Two other participants commented that they are used to using iPhones and were confused by the different keyboard layouts in the tablet version of LINE. Another participant mentioned that preferences may vary from person to person as follows:

*“Maybe it would be better if patients could choose whether to answer on a tablet or verbally.* *If it is only available on tablets, patients who are not familiar with using tablets may panic.* *Sometimes, the elderly are not the only ones who have that tendency.* *However, I think there are a lot of people who would still find it useful.”*

#### Category: Concern

This category was classified into the following two subcategories: information security management and poor network condition.

Regarding information security management, comments were obtained from three participants who were concerned about the possibility of information leakage, as follows:

*“I wonder where my answers will go in the LINE network. I use it on a regular basis, and I am sometimes concerned about the security of LINE. So, I worry about leakage of my private information.”*

Regarding the network condition, comments were obtained from two participants who were concerned that the responses of the chatbot might be delayed, which may lead to communication errors with the chatbot.

#### Category: Benefit

This category was classified into the following seven subcategories: superior ease of use, effective use of waiting time, reduced time required for medical interview, overall efficiency of the clinical experience, expanded preliminary screening coverage, research use, and safety in the coronavirus disease 2019 pandemic.

Five participants commented that this system is more convenient than face-to-face preliminary screening. The following are some of the representative reasons.

*“The chatbot system seems easier for communication than talking face-to-face. I think younger people would prefer this system. Just like I play video games.”*

*“If this system could be operated at home, it would be easier for cancer patients. It is difficult to come to the hospital frequently when a patient is in bad condition due to cancer treatment.”*

*“When you first meet a medical professional, you get nervous, don’t you? I get nervous when I see someone in a white coat and wonder what they are going to talk about, but when it comes to the chatbot, I think I can operate it in a more relaxed manner.**”*

Five participants commented that they could make effective use of their time if they could use the system while waiting for medical examinations, and two participants commented that the system would enable faster preliminary screening than face-to-face screening. One participant made the following comment, which envisioned advantages such as the ability to utilize the system in areas where there are no specialists in genetic medicine:

*“I still think there is a small number of experts specializing in genetic medicine, and in some areas of Japan, there are none. I think the advantage of this system is that it can meet the needs of genetic medicine by allowing preliminary screening in such areas.”*

#### Category: Implementation

This category was classified into the following two subcategories: implementability and considerations during operation.

Regarding the implementability of this system for clinical application, eight participants commented that it had excellent feasibility; some comments stated that they could not think of any particular problem to be considered for clinical application for the following reasons:

*“I don’t think that I need any more consideration or ingenuity.* *The questions from the chatbot were also what was explained to me at the beginning by the CGC, so I was not asked anything unexpected by the chatbot.* *LINE is familiar to people in my age group, in their 50s, so I think it is easy to operate.”*

*“If you gave me a tablet in the outpatient department, what I would do would be the same as a paper-based medical questionnaire.* *People who are not used to operating tablets may need in-person support, but once they get used to it, they can input the data at once.”*

However, six participants provided points for improvement. The most representative of these comments came from three participants who mentioned the need for in-person support in situations where system errors occur or information cannot be fully entered using the system. Moreover, two participants commented that the system might be easier to understand if voice guidance was provided, while two participants commented that an explanation by medical staff before using the system was necessary. Additionally, one participant commented that she would like to receive some form of feedback on how her risk status was evaluated based on the information she entered.
